# Delayed post-traumatic Tension Hydropneumocephalus; a Case Report of Conservative Treatment 

**DOI:** 10.22037/aaem.v9i1.1172

**Published:** 2021-02-27

**Authors:** Talayeh Mirkarimi, Ehsan Modirian, Peyman Namdar, Mohammad Salek

**Affiliations:** 1 Emergency Department; Rajaei Hospital, Medical Faculty, Qazvin University of Medical Science, Qazvin, Iran.

**Keywords:** Pneumocephalus, Craniocerebral Trauma, Conservative Treatment, Case Reports

## Abstract

Pneumocephalus refers to the presence of air in the cranial cavity. Trauma is the most common cause of acquired pneumocephalus. Tension pneumocephalus occurs when intracranial accumulation of air causes high pressure on the brain as compared to extracranial pressure. Tension pneumocephalus is usually acute, and causes neurological symptoms, and its delayed form rarely occurs. A 12-year-old girl presented with a headache, lethargy, mild fever, and nausea from two days before admission to emergency department of Shahid Rajaei Hospital, Qazvin, Iran. The patient had a history of head trauma in a driving accident six weeks before and had undergone brain computed tomography (CT) scan in another centre, which had revealed no sign of pneumocephalus. The patient had been treated for one week and had been discharged in good general condition.

Considering her reduced consciousness, the patient underwent brain CT scan again in our centre. CT scan revealed tension hydropneumocephalus. The patient was transferred to the intensive care unit (ICU) for treatment. Considering the trend of her recovery, the patient was a candidate for conservative non-surgical therapy based on the in-charge neurosurgery specialist’s decision. The patient reported no complications during the six-month follow-up. Delayed tension pneumocephalus is among neurosurgery emergencies usually treated with early surgical intervention and dura defect restoration, but this patient received non-surgical treatment without any serious problem during the six-month follow-up.

## Introduction

Pneumocephalus refers to a pathologic intracranial accumulation of air, and is categorized as epidural, subdural, subarachnoid, intra-parenchymal, and intra-ventricular types (1). Head and facial trauma is the most common cause of pneumocephalus and is responsible for 75% of cases (2). Other factors that can cause pneumocephalus include otitis media, skull base tumours, neurosurgical procedures (3), anaesthesia with nitric oxide, positive pressure ventilation, hyperbaric oxygen therapy, barotrauma, spinal anaesthesia, Intracranial Pressure (ICP) monitoring, intraoperative infusion of mannitol (4, 5), and gas-forming infections in the central nervous system (CNS). Moreover, spontaneous form has been rarely reported (3). Intracranial accumulation of gas can be acute (<72 hours) or delayed (>72 hours). Pneumocephalus is also divided into simple and tension types. Tension pneumocephalus refers to the type that causes higher pressure on the brain parenchyma compared to extracranial pressure (6-8). Since this type can cause neurological disorders that are potentially life-threatening, such as cerebral herniation, its early diagnosis is highly important (8). Here we present the case of a 12-year-old girl who presented with headache, lethargy, mild fever, and nausea from two days before admission to emergency department and history of head trauma 6 weeks before. She was diagnosed with delayed tension hydropneumocephalus and treated by conservative management without any problem during the 6-month follow-up. 

## Case presentation

A 12-year-old girl presented to emergency department of Shahid Rajaei Hospital in Ghazvin, Iran, with headache, drowsiness, mild fever, nausea, vomiting, and lethargy form two day before admission. The patient had a history of head trauma in a driving accident six weeks before and had been hospitalized in Hamedan city, where she had undergone spiral brain computed tomography (CT) scan without contrast due to reduced consciousness. Brain CT scan reported right frontal contusion, right frontal bone fracture involving frontal sinus and filling of ethmoid sinuses. The patient had received treatment in that centre for one week and was discharged in good general condition. The patient did not report any history of seizures, otorrhoea, or rhinorrhoea during these six weeks. 

The presenting vital signs of the patient were as follows: pulse rate: 88/minute, respiratory rate: 18/minute, blood pressure: 105/75 mmHg, axillary Temperature: 37.8°C, Glasgow coma scale (GCS): 13/15 (eye response = 3, motor response = 6, verbal response = 4). Pupils were symmetrical of 3 millimetres and reduced response to light was detected in the right pupil. The four limbs had equal force of 5/5. Deep tendon reflexes (DTRs) were 2+ and symmetrical, and bilateral plantar reflex was symmetrical and downward. Other clinical examination findings were unremarkable.

**Figure 1:**
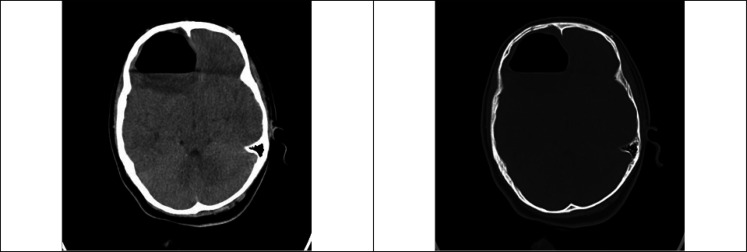
Spiral brain CT scan without contrast (axial cut) revealed air-fluid level in the frontal parenchyma with midline shift and compression effect on the anterior horn of the lateral ventricles in the right frontal lobe.

**Figure 2:**
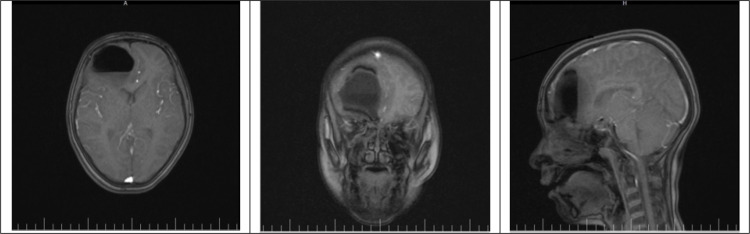
Magnetic resonance imaging (MRI) with contrast (Axial, coronal and sagittal views) revealed no rim enhancement around the lesion, which ruled out brain abscess and confirmed tension hydropneumocephalus diagnosis

The patient underwent brain CT scan again in our centre, which revealed hydropneumocephalus in the frontal parenchyma with midline shift and compression effect on the anterior horn of the lateral ventricles in the right frontal lobe (figure 1). Thus, the patient underwent treatment with phenytoin and 100% concentration oxygen. Moreover, given her mild fever and likelihood of brain abscess, a broad-spectrum antibiotic (Vancomycin) was administered until magnetic resonance imaging (MRI) was done. Neurosurgery emergency consultation was requested, and the patient was admitted to the Intensive care unit for further treatments. Considering the trend of recovery and at the neurosurgeon’s discretion, the patient was a candidate for conservative non-surgical therapy. MRI with contrast on the third day of admission revealed no rim enhancement around the lesion, and confirmed the diagnosis of tension hydropneumocephalus (figure 2). Therefore, antibiotic was discontinued. On the seventh day, the patient was advised to continue taking oral phenytoin, and was discharged in good general condition. The patient reported no complications during the six-month follow-up. 

## Discussion

Two main theories usually explain the mechanism of developing tension pneumocephalus: 1) the ball-valve theory, in which air enters the skull unilaterally, but cannot leave (9, 10). 2) Inverted soda valve bottle theory, in which, air is drawn into the skull by the negative pressure created due to reduced cerebrospinal fluid (CSF) volume (for whatever reason)(4, 5, 11). Headache is the most common symptom of pneumocephalus (12). The clinical presentation of tension pneumocephalus includes headache, generalized seizure, agitation, delirium, abnormal reflexes, changes in consciousness level, and changes in pupil size and response. Tension pneumocephalus can mimic the manifestations of an intracranial space occupying lesion, and can lead to signs of brainstem displacement, including changes in respiratory rhythm and cardiac arrest, if it occurs in the posterior cranial fossa (13). 

Brain CT scan is the gold standard for diagnosis of tension pneumocephalus, which can also be diagnosed using plain radiography (3). The typical pathognomonic view of pneumocephalus is referred to as “Mount Fuji sign”, which is described as bilateral subdural hypoattenuation with compression and detachment of the frontal lobes (14, 15). Generally, most cases with pneumocephalus need conservative treatment. Simple pneumocephalus with no neurological signs is treated by head elevation, administration of osmotic diuretics, analgesics and antipyretics, and also preventing manoeuvres that increase intracranial pressure such as the Valsalva manoeuvre (2, 5). High concentration oxygen increases absorption of pneumocephalus. Antibiotics are recommended if meningitis is suspected (3). In cases with tension pneumocephalus with substantial intracranial pressure, emergent decompression is indicated (2, 5, 16). Once air is aspirated, closure of dural defect is the only certain way to prevent recurrence of tension pneumocephalus (8). 

This case had several important points worth discussing. The patient’s brain CT scan showed tension pneumocephalus with air-fluid level, which is extremely rare (17). The patient had no external lacerations after the initial trauma and reported no history of posttraumatic rhinorrhoea or otorrhoea. In this patient, tension pneumocephalus can probably be explained in the context of occult dural laceration fracture of the right frontal sinus and ethmoid sinus walls, which let air enter unilaterally (Ball-valve mechanism). Moreover, given the rare brain CT scan view and mild fever, an intracranial abscess (secondary to gas-forming organisms) is a highly important differential diagnosis, which explains why a broad-spectrum antibiotic was administered for the patient until MRI was performed. 

## Conclusion:

Delayed tension pneumocephalus is a neurosurgical emergency and a complication rarely seen after head trauma, which requires prompt surgical intervention. However, depending on size and severity of signs and symptoms some cases could be managed conservatively and with long-term follow-up.
